# A Genomic Method for Combating Wildlife Trafficking: SNP-Based Traceability of Four Endangered Species in China

**DOI:** 10.3390/ani16071052

**Published:** 2026-03-30

**Authors:** Jilai Zhao, Xibo Wang, Yang Teng, Paul A. Garber, Huijuan Pan, Jiwei Qi

**Affiliations:** 1 School of Ecology and Nature Conservation, Beijing Forestry University, Beijing 100083, China; 2 State Key Laboratory of Animal Biodiversity Conservation and Integrated Pest Management, Institute of Zoology, Chinese Academy of Sciences, Beijing 100101, China; 3 Ministry of Education Key Laboratory for Biodiversity Science and Ecological Engineering, College of Life Sciences, Beijing Normal University, No. 19 Xin Jie Kou Wai Avenue, Beijing 100875, China; 4 International Centre of Biodiversity and Primate Conservation, Dali University, Dali 671003, China; p-garber@illinois.edu; 5 Department of Anthropology and Program in Ecology, Evolution, and Conservation Biology, University of Illinois, Urbana, IL 61801, USA

**Keywords:** wildlife trafficking, forensic identification, Tibetan macaque, eared pheasant, Chinese pangolin, single-nucleotide polymorphism (SNP), population traceability

## Abstract

Wildlife trafficking poses a severe threat to global biodiversity and ecosystem stability, necessitating robust forensic tools for tracing the origins of illegally traded taxa. In this study, we developed a method of single-nucleotide polymorphism (SNP)-based molecular markers to enable precise geographical traceability of four animal species native to China: the Tibetan macaque (*Macaca thibetana*), brown eared pheasant (*Crossoptilon mantchuricum*), blue eared pheasant (*Crossoptilon auritum*), and Chinese pangolin (*Manis pentadactyla*). We studied these four species because their DNA is characterized by distinct population genetic structure, they are subjected to illegal trafficking, and given their diverse evolutionary histories, this allowed us to assess the general applicability of our forensic genetic framework in reducing wildlife crime. Based on whole-genome resequencing data from 26 Tibetan macaques, 51 eared pheasants and 42 Chinese pangolins, we performed population genetic analyses to elucidate their genetic structure and identify population-specific loci. The results indicated that all samples from these four species showed clear genetic differentiation and distinct clustering, allowing us to design primers to facilitate PCR-based traceability. We also assessed the utility of mitochondrial DNA (mtDNA) for tracing Tibetan macaques and both species of eared pheasants. We found that traceability accuracy using mtDNA was lower than when using SNPs. Our research offers a SNP-based traceability framework that accurately determines the geographical origin of wildlife samples to the genetic population level, and this provides a powerful tool for combating illegal trade and aiding conservation efforts.

## Simple Summary

Illegal wildlife trade threatens biodiversity and undermines conservation efforts, particularly for endangered species with fragmented populations. Identifying the geographic origin of confiscated wildlife products is therefore critical for law enforcement and conservation management. In this study, we developed a single-nucleotide polymorphism (SNP)-based genetic traceability framework for four endangered animal species in China: the Tibetan macaque, brown eared pheasant, blue eared pheasant, and Chinese pangolin. Using genome-wide SNP data, we identified informative genetic markers capable of distinguishing population origins with high accuracy. Our results demonstrate that SNP-based approaches provide a reliable and broadly applicable tool for tracing the geographic provenance of illegally traded wildlife. This method can support judicial decision-making, strengthen wildlife law enforcement, and contribute to the long-term conservation of threatened species.

## 1. Introduction

Wildlife trafficking is a global conservation issue, with devastating consequences for the environment, the loss of biodiversity, and can trigger an invasion of non-native species leading to the spread of infectious diseases [1]. This illicit trade has grown rapidly in scale and complexity alongside transnational smuggling networks, and its significant economic magnitude has led to its recognition in policy and research as a major form of transnational crime [2,3]. Poaching for illegal trade contributes to the threat of local population extinction to many endangered wildlife species. For instance, pangolins (*Manis* spp.) and western black rhinoceroses (*Diceros bicornis longipes*), whose scales and horns are often used in traditional medicine, are extensively poached and hunted, pushing them to the verge of extinction [4,5]. Wildlife trafficking occurs both within and across national borders relying on both local and international syndicates complicating law enforcement, and making the collection of admissible evidence and the determination of specimen origin particularly challenging.

Given changes in China’s social and environmental national development agenda beginning in 2012, substantial progress has been achieved in promoting biodiversity conservation through strengthened legal frameworks, conducting nationwide biodiversity assessments, engaging in large-scale ecological restoration programs, expanding protected-areas, and monitoring wildlife trafficking networks [6,7,8]. Nevertheless, wildlife crime remains prevalent in China, and current judicial practices have often failed to enforce laws and guard against the ecological and public-health risks associated with wildlife exploitation, underscoring the need for more comprehensive legislation and stronger evidentiary support [9]. Although recent revisions to the Wildlife Protection Law have shifted sentencing criteria from the “number of wildlife involved” to the “economic value of wildlife involved,” the valuation system remains overly simplistic, often applying uniform pricing across species within the same taxonomic group, and thereby underestimating the conservation value of threatened taxa [10]. In addition, limited knowledge of rare species and underdeveloped systems of wildlife source tracing and individual identification continue to limit the implementation of science-based conservation policies to deter wildlife trafficking [11].

Wildlife forensics is needed to address five key issues in biodiversity conservation: species identification, geographical origin tracing, individual identification, distinguishing between captive and wild individuals, and developing recommendations to protect threatened species [12]. Traditional forensic approaches have relied heavily on morphological characteristics for species identification, which can be effective when intact specimens are available [13]. However, morphological methods are highly dependent on expert experience, difficult to standardize, and often unreliable when specimens are incomplete, processed, or degraded [14]. However, over the past few years, advances in molecular genetics have transformed wildlife forensics, enabling more accurate and reproducible identification based on DNA evidence [15,16,17].

Early molecular approaches to wildlife identification primarily relied on short tandem repeats (STRs) and mitochondrial DNA (mtDNA) markers for species identification [18]. However, despite their widespread application, these markers present several inherent limitations, including susceptibility to DNA degradation, maternal inheritance restricting lineage tracing, heteroplasmy and nuclear mitochondrial pseudogenes (numts) that may compromise sequencing accuracy, and limited resolution for fine-scale geographic assignment [16,19,20,21]. Moreover, the lack of standardized protocols, quality assurance systems, and inter-laboratory validation often has resulted in limited acceptance of genetic evidence derived from these markers [22].

Single-nucleotide polymorphisms (SNPs), as third-generation molecular markers, offer several advantages for wildlife forensic applications. SNPs are abundant across the nuclear genome, exhibit high genotyping stability, and can be reliably amplified from short DNA fragments, making them particularly suitable for the analysis of degraded forensic samples [23,24]. Moreover, genome-wide SNP data have demonstrated strong potential for high-resolution population assignment and traceability in food authentication, livestock management, and wildlife conservation [25,26,27]. Despite this promise, SNP-based forensic traceability frameworks remain underdeveloped for most endangered wildlife species, particularly at the subspecies or population level.

A prerequisite for implementing population-level genetic traceability is the delineation of biologically meaningful and genetically distinguishable populations within a species. Only after such traceable units are defined can population-specific genetic markers be identified and applied to forensic investigations. This requirement is especially relevant for species with complex evolutionary histories, fragmented distributions, or ongoing gene flow, where coarse taxonomic identification provides limited enforcement value [28,29]. In this study, we establish a SNP-based forensic traceability framework using whole-genome resequencing data from four species native to China: the Tibetan macaque (*Macaca thibetana*, *Near Threatened*), the brown eared pheasant (*Crossoptilon mantchuricum*, *Least Concerned*), the blue eared pheasant (*C. auritum*, *Least Concerned*), and the Chinese pangolin (*Manis pentadactyla*, *Critically Endangered*). These four species were selected for study because (i) each exhibits pronounced population genetic structure, (ii) they are frequently involved in wildlife crime (China Judgements Online, https://wenshu.court.gov.cn/), and (iii) each represent distinct evolutionary histories and taxonomic groups, allowing assessment of the general applicability of the proposed framework to reduce wildlife trafficking.

The Tibetan macaque (*Macaca thibetana*), which is widely distributed across southern China, comprises four recognized subspecies [30]. In recent years, increasing anthropogenic pressure and habitat degradation have resulted in declines in both its distributional range and population size, which is estimated to be approximately 20,000 individuals [31,32,33]. Eared pheasants (*Crossoptilon* spp.) include four distinct species. The brown eared pheasant (*C. mantchuricum*) occurs in three regions of north-central China [34], where its population has declined over the past five decades in response to deforestation and overhunting [35]. Its current population is estimated to be approximately 25,000 [36,37,38]. The blue eared pheasant (*C. auritum*) is distributed in central-western China [39]. It is the sister species to the brown eared pheasant, with an estimated divergence time of approximately 0.3 million years ago. The blue eared pheasant is also experiencing a population decline; however, estimates of its current population size remain unavailable [40]. Finally, the Chinese pangolin (*Manis pentadactyla*) is distributed across the 12 southern provinces/regions of China and comprises three recognized subspecies [41,42]. Decades of illegal hunting and habitat loss have posed severe threats to its survival [43], leading to a continuous contraction of its distributional range [44]. In 2008, the official estimated population size of the Chinese pangolin (*Manis pentadactyla*) in China was 64,000 individuals. However, an evaluation by Zhang et al. (2010) indicated that the Chinese population was between 25,000 and 50,000 individuals [45]. There is evidence that the species has likely been extirpated from several provinces such as Henan and Jiangsu, and at present only sporadic sightings have been recorded [45,46].

In order to construct a suitable framework for identifying and tracing trafficked wildlife specimens, we downloaded the genome resequencing data of 26 Tibetan macaques, 51 eared pheasants and 42 Chinese pangolins. Using genome-wide SNP data, we first delineated traceable genetic populations within each taxon through population structure analyses. We then identified population-specific SNP loci and developed PCR-amplifiable markers suitable for forensic application. Finally, we evaluated the performance of SNP-based traceability by comparing mitochondrial DNA-based approaches. Together, this work provides a practical and transferable genomic framework for population-level wildlife traceability, with direct implications for law enforcement and biodiversity conservation across China and worldwide.

## 2. Materials and Methods

### 2.1. Sample Collection and Downloading

**Figure 1:**
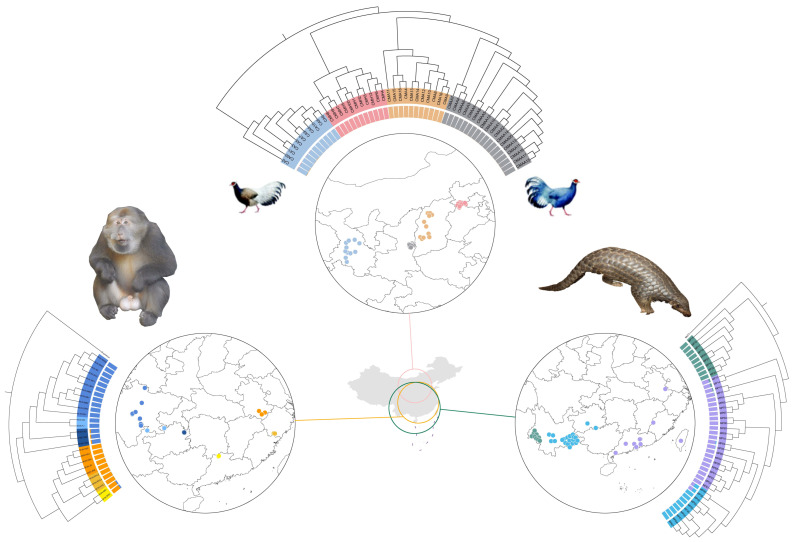
Sampling location of the four animal species used in this study, together with the plots of phylogenetic tree and admixture analysis. The sampling location of Tibetan macaques (**left**), two species of eared pheasants (**up**), and Chinese pangolins (**right**), surrounded by their phylogenetic tree (run with RAxML) and admixture analysis with the lowest cross-validation (CV) error.

We downloaded whole-genome data of 26 Tibetan macaque from a previously published research project (GSA: PRJCA026025) [33]. All samples were collected from southern provinces of China, including Sichuan, Hunan, Anhui, Guizhou, Yunnan, and Zhejiang (Figure 1). These six provinces encompass the core distribution range of Tibetan macaque in southern China, which covers the two major genetic clades (eastern and western). In the case of eared pheasants, we decided to combine genetic samples from both species due to the fact that our sample only included 11 blue pheasants, all from the same reserve in Gansu province. We regarded them as a single genetic population. The data for brown eared pheasants represent a total of 40 individuals (11 from Shaanxi Province, 18 from Shanxi Province, 11 from Hebei Province and Beijing). All samples were from Bioproject PRJNA1057913 [47]. (Figure 1). In addition, genetic data from a total of 42 Chinese pangolin samples were downloaded, including 23 individuals from the National Center for Biotechnology Information (NCBI) (22 from BioProject PRJNA529540 and one from PRJNA20331) and 16 individuals from a study by Wei et al. (2024) [48]. One Malayan pangolin (*M. javanica*) sample from NCBI (BioProject PRJNA529540) was included as an outgroup.

### 2.2. Data Filter and Variant Detection

For Tibetan macaques, we downloaded the reference genome “Mmul_10” from NCBI (GCF_003339765.1). For eared pheasants, one female *C. mantchuricum* was used as a reference genome. For Chinese pangolins, we used a reference genome from the Chinese National GeneBank database (CNGBdb) (BioProject: PRJCA020583).

We performed variant calling using the Genome Analysis Toolkit (GATK v4.1.5.0, Broad Institute, https://gatk.broadinstitute.org). Individual gVCF files were generated with HaplotypeCaller, then merged into a single VCF file using the CombineGVCFs tool implemented in GATK v4.1.5.0.

In order to remove the low-quality and high-missing-rate loci, we used “VariantFiltration” and tools from “vcftools” based on the following criteria: QD < 2.0 || MQ < 40.0 || FS > 60.0 || HaplotypeScore > 13.0 || MQRankSum < −12.5 || ReadPosRankSum < −8.0. Finally, vcftools were employed for additional filtering with parameters: “--maf 0.05 --max-missing 0.9”.

### 2.3. Population Genetic Structure Analysis

We used RAxML v8.2.12 [49] to construct a maximum-likelihood phylogenetic tree. One Barbary macaque (*M. sylvanus*) sample downloaded from the NCBI (SRA: SRR14843527) was used as an outgroup for Tibetan macaques. Website iTOL (itol.embl.de/) [50] was used for visualization of the phylogenetic trees. To assess population structure, we performed principal component analysis (PCA) and calculated pairwise genetic relatedness using Normalized identity-by-state (IBS) kinship in VCF2PCACluster v1.40 [51]. Kinship analysis was included to detect potential close relatives or family structure that could otherwise bias downstream clustering and marker selection. Genetic ancestry was inferred with ADMIXTURE v1.3.0 [52], testing K = 2–6 with 40 bootstrap replicates and selecting the optimal K based on the lowest cross-validation error. Gene flow among populations was evaluated using Dsuite v0.5 [53] for calculating D and f4-ratio statistics.

### 2.4. Gene Screening, Specific Loci Filtering and Primer Design

**Figure 2:**
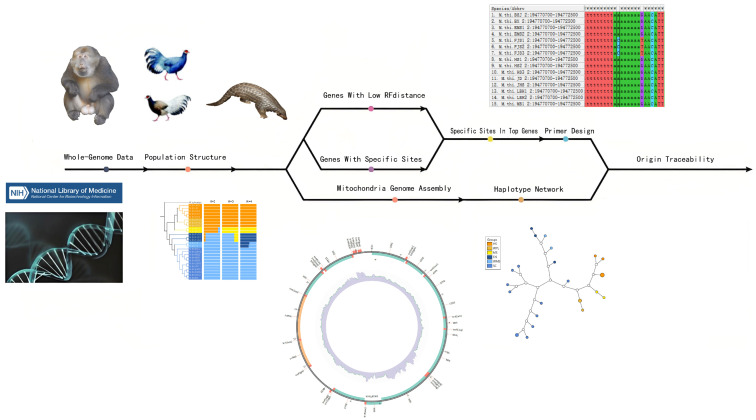
Procedure of Traceability using Tibetan macaques as example. The complete traceability pipeline used in this research, and all the example figures are based on the results from an analysis of Tibetan macaques. The image located in the upper right corner shows the genomic sequences for Tibetan macaques from the site of FJS population, and the mutations are shown in (Table 1). Images located in the bottom center are phylogenetic trees based on admixture, mtDNA assembled with MitoZ, and the haplotype network of mtDNA.

Population-specific primers require identification of short, phylogenetically informative gene fragments. To achieve this, we first extracted the longest transcript (>2000 bp) of each gene from exon regions using bcftools consensus (Figure 2). For each gene, we reconstructed a gene tree with RAxML and calculated its topological distance to the species tree using the Robinson–Foulds (RF) metric. Pearson correlation analysis was then applied to test the relationship between gene length and RF distance (R = −0.21, *p* < 2.2 × 10^−16^; Figure S4), as longer genes are statistically more likely to retain strong phylogenetic signal and were therefore prioritized for primer design.

**Table 1:** Primer information of Tibetan macaques.

Pop	Gene	Mutation	Left Primer	Right Primer
FJS	ROBO2	A-C	TATCAGAGGAGGGATCAAATGA	GTTAGGGACCTGAAGAAAGATG
HS	FMN2	G-A	ACTTTTGCAGATGGATATGGAA	TTCAGAAATGTTTTGAATGCCC
MS	ROBO2	A-G	TGACACGTAGATCCATACCATA	TATTCATTTGGGGAAGGGAGTA
SC	ARL6	G-T	CTGACTTCATAAACTCTGCACT	GAGGTGAGGAAGGAATTCAATT
WMS	RGSL1	C-T	GATCAGAGAGGTTACAGTTGTC	CAAAGGGGATGATAGCGGAG
WYL	ABCA13	T-C	ATTCAGCTATTGGAAATCTGCA	ACATGTTGATTGGTGTGAGTTA

We used “bcftools consensus-I” to extract fna files from VCFs according to the General Feature Format (GFF) file. Due to the phenomenon of alternative splicing, a single sequence may produce multiple transcripts [54]. Since exon regions encode protein-coding functions and are more strongly subject to selective pressures compared to intronic regions [55], we focused on fixed sites located within genic regions in this study. The longest transcript of each gene was identified. We selected genes if their longest transcript exceeded 2000 bp for primer design and amplification. Transcript segments of the same gene from all samples were merged and gene trees were constructed using “raxmlHPC-PTHREADS”. The “raxml-ng” command with the parameter “--rfdist” was used to calculate the Robinson–Foulds (RF) distance [56] between the species tree and all gene trees. Genes with relatively small RF distances were regarded as target genes.

The VCF files were split according to group and generated by population structure analyses. Sites were then filtered based on a homozygous rate threshold specific to each group. For the pheasants, a site was picked only if 100% of the individuals were homozygous for that variant. For the Tibetan macaques, we picked sites with a Homozygous Allele Frequency (HAF) ≥ 80%, and for Chinese pangolins 90%. The use of different HAF thresholds reflected differences in sample size and population genetic characteristics among taxa. Macaques exhibit relatively recent divergence, rapid radiation, and signatures of incomplete lineage sorting [57], which may reduce the proportion of fully fixed sites across populations. Therefore, a slightly lower threshold (80%) was adopted to retain sufficient informative loci. In contrast, Chinese pangolins show clearer population differentiation and stronger genetic structure, allowing the application of a more stringent threshold (90%) to ensure high specificity of population-specific markers. For each population, the homozygous sites were intersected with the opposite homozygous sites from all other populations. The resulting sites were defined as population-specific sites. Identified genes contained population-specific sites from the GFF. Subsequently, these genes were intersected with the target genes, and the final gene set obtained from this intersection was plotted using RectChr (v1.41) (https://github.com/BGI-shenzhen/RectChr, accessed on 27 March 2026).

We selected appropriate sites from the RectChr schematic, cut 900 bp of flanking sequence on both sides, and extracted the whole sequence (1801 bp in total). These sequences were used for primer design with the Primer3 (version 4.1.0) and Primer3Plus (https://primer3.ut.ee/, https://www.primer3plus.com/). Parameters were set to: primer size, min 20, opt 22, and max 26; primer Tm, min 52, opt 56, and max 62; primer GC%, min 30, opt 50, and max 70.

### 2.5. MtDNA Assembly and Haplotype Network Construction

The de novo assembly was made for the mitochondrial genome of Tibetan macaques and eared pheasants from raw reads using MitoZ (v 3.6) [58]. Command “muscle” was used to align the mitochondrial genome. We used fastHaN (v1.0) [59] to construct the haplotype network, and visualized the result with tcsBU (v1.0) [60]. In all statistical analyses, *p* was set as ≤0.05.

## 3. Results

### 3.1. SNP Calling, Variants Detection and Traceable Genetic Population Identification

For Tibetan macaques, the average sequencing depth was 26.50X (20.45X–40.3X) with a mapping rate of 99.24% (97.92–99.71%). A total of 26,094,572 SNPs were identified following quality control and filtering (Table S1). Regarding eared pheasants, the average resequencing coverage reached 19.0X (13.4X–43.2X), the average mapping rate was 97.3% (83.6–98.9%), and a total of 2,257,752 SNPs were detected (Table S2). In the case of Chinese pangolins, the average coverage was 30.32X (11.84X–50.75X), with a mapping rate averaging 97.87% (70.81–99.94%), and 1,394,113 SNPs were identified (Table S3).

**Figure 3:**
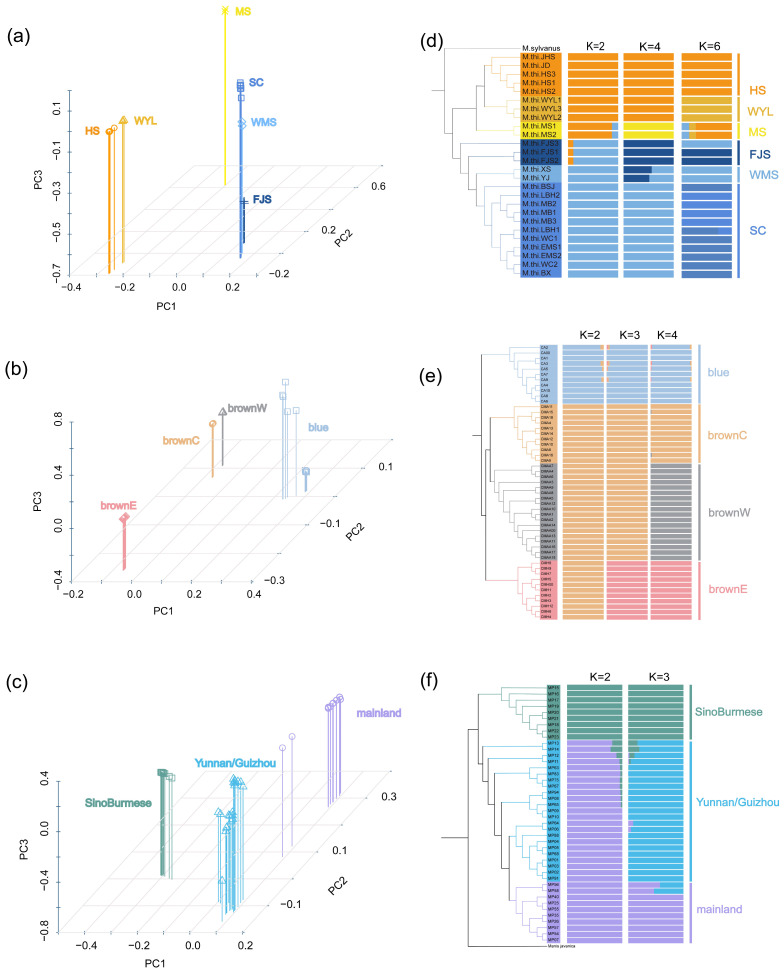
(**a**) The PCA plot with the three principal components for Tibetan macaques. Tibetan macaque samples formed several distinct and tightly grouped clusters. (**b**) The PCA plot with the three principal components for eared pheasants. The clusters for eared pheasants were tight, but PC2 separated the brownE from all other populations. (**c**) The PCA plot for the three principal components for the Chinese pangolin. Except for the SinoBurmese populations, the cluster of other two populations did not indicate a tight structure. In particular, two individuals from the Yunnan/Guizhou population had a distant relationship from the other populations as measured by PC2. (**d**) Admixture analysis of Tibetan macaques with a K of 2, 4, 6. The CV error reached its minimum when K = 2. The relative length of each colored segment corresponds to the proportional contribution from a distinct ancestral population. (**e**) Admixture analysis of eared pheasants with a K of 2, 3, 4. The CV error reached its minimum when K = 4. (**f**) Admixture analysis of Chinese pangolins with a K of 2 and 3. The CV error reached its minimum when K = 3.

**Figure 4:**
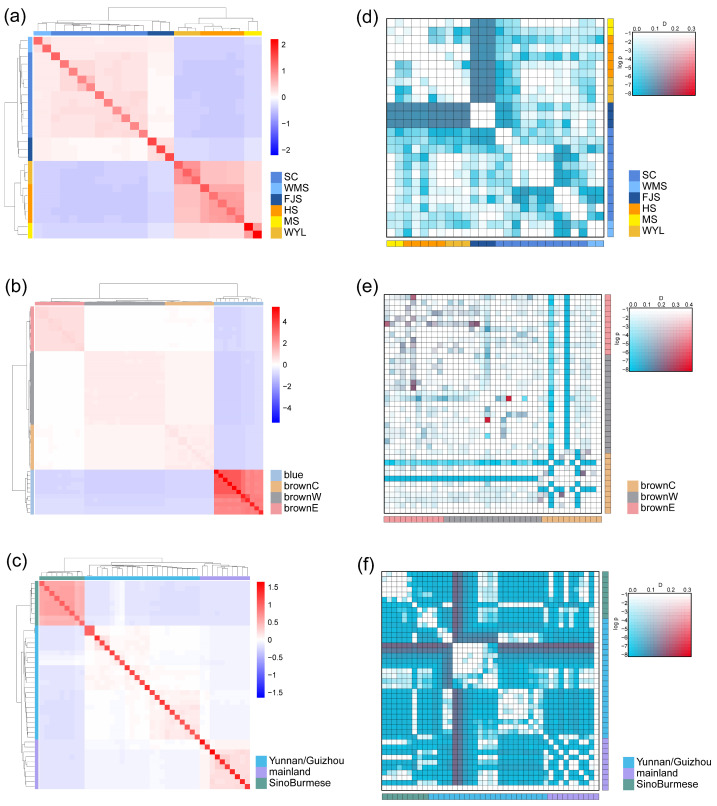
Results of the Kinship using Normalized_IBS and gene flow between individuals. (**a**) Kinship matrix of Tibetan macaques using Normalized_IBS method. Heatmap coloration (red scale) indicates genetic relatedness, with deeper red colors representing closer relationships. The diagonal of the matrix represents auto-relationships (each individual vs. itself). (**b**) Kinship matrix of eared pheasants using the Normalized_IBS method. Due to the closer genetic relationships among the three brown eared pheasants and their relatively distant relationship with the blue eared pheasant, the blue eared pheasants appear deeper color, while individuals within the brown eared pheasant population appear lighter in color. (**c**) Kinship matrix of Chinese pangolins using the Normalized_IBS method. Individuals in the SinoBurmese populations appeared to have a closer genetic relationship compared to pangolins in the other two groups. (**d**) Gene flow matrix of Tibetan macaques generated by Dsuite Dtrios. Matrix shows the asymmetry of gene flow between all individuals with the Barbary macaque as the outgroup. Color intensity (from blue to red) encodes the strength of gene flow/D-statistic values, warmer (redder) colors suggest stronger asymmetrical gene flow. The transparency scale represents −log_10_ (*p*-value), where higher opacity indicates greater statistical support. (**e**) Gene flow matrix of brown eared pheasants generated by Dsuite Dtrios. Due to the lack of a suitable outgroup, we selected the blue eared pheasant as the outgroup for the brown eared pheasant. (**f**) Gene flow matrix of Chinese pangolins generated by Dsuite Dtrios. The matrix revealed a wide range of asymmetrical gene flow in the three populations, with strong statistic support. A Malaysian pangolin was chosen as the outgroup.

The phylogenetic tree of Tibetan macaques revealed that the 26 individuals could be divided into two major clades, eastern and western, with each major clade comprising three distinct subclades. The eastern clade included individuals from three areas Huangshan (HS), Wuyanling (WYL), and Mangshan (MS), while the western clade comprised individuals from Sichuan (SC), Fanjingshan (FJS), and Wumengshan (WMS) (Figure 3d). This division was supported by PCA and Admixture analyses. Kinship analysis also suggested that the eastern and western clades shared a distant phylogenetic relationship (Figure 4a). In the admixture analysis, the cross-validation (CV) error reached its minimum at K = 2 (Figure 3d and Figure S1). In the PCA results, PC1 distinguished the eastern and western clades, and PC1 and PC2 together distinguished all six populations (Figure 3a). The Dsuite result showed asymmetric gene flow between the FJS population and all eastern populations, which provides additional insight into the phylogenetic history of western Tibetan macaques (Figure 4d).

The phylogenetic analysis identified four distinct genetic clusters: one corresponding to the blue eared pheasant and three corresponding to geographically separated populations of the brown eared pheasant. Within the brown eared pheasant clade, the brownW population was a sister clade of the brownC population, and collectively they formed a sister clade to the brownE population (Figure 1). The ADMIXTURE analysis yielded the lowest cross-validation error at K = 4 (Figure 3e and Figure S2). In the PCA results, PC1 (55.01%) separated the blue eared pheasants from the brown eared pheasants. PC2 (10.05%) separated the brownE population from all other populations (Figure 3b). Kinship analysis further revealed significantly higher genetic relatedness within the brownE pheasant population compared to brownW and brownC populations. Collectively, these results suggest that early geographical isolation might have driven the divergence of the brownE pheasant population (Figure 4b).

For the Chinese pangolins, the phylogenetic tree revealed that the Yunnan/Guizhou population formed a subclade nested within the mainland clade. Together, these two populations constituted a sister clade to the SinoBurmese clade (Figure 1). The ADMIXTURE analysis indicated the lowest cross-validation error at K = 3 (Figure 3f and Figure S3). In the PCA, PC1 separated the SinoBurmese population from the other two populations. PC2 segregated the mainland population from the other two groups, while two individuals from the mainland population were positioned distantly from the mainland cluster (Figure 4c). All pangolin genome data analyzed in this research are from a study by Wei et al. (2024) [48]. These authors reported three genetic clusters of Chinese pangolins: MPA, MPB (MPB1, MPB2), and MPC. The SinoBurmese pangolins belong to MPA; mainland pangolins belong to MPB2; and the Yunnan/Guizhou pangolins belong to MPB1. The Dsuite analysis revealed widespread and statistically significant asymmetrical gene flow among the three populations, which indicates the possibility of introgression during their evolutionary history (Figure 4f).

### 3.2. Traceable Gene Screening, Specific Sites Filtering and Primer Design

Genes whose longest transcript exceeded 2000 bp were filtered from exon regions. For Tibetan macaques, 5675 genes were examined and for eared pheasants, 4373 genes were examined. Phylogenetic trees were constructed using these genes, and the Robinson–Foulds (RF) distance between each gene tree and the species tree was calculated. Genes were then ranked based on their RF distance scores. Pearson correlation analysis revealed a significant negative correlation between gene length and RF distance (R = −0.21, *p* < 2.2 × 10^−16^) (Figures S4 and S5).

The filtering results from Tibetan macaques revealed the following numbers of fixed sites: 17,702,131 for FJS, 21,672,273 for HS, 20,370,286 for MS, 9,571,350 for SC, 20,307,376 for WMS, and 22,227,667 for WYL. These sites were realigned to the genome annotation file (GFF) to identify their respective genes. By intersecting the genes containing these sites with those previously shown to have low RF distances (Figure S4), five key genes were identified: ROBO2 for FJS (194,771,600 bp, A-C) and MS (194,231,893 bp, A-G), FMN2 for HS (8,614,692 bp, G-A), ARL6 for SC (178,123,035 bp, G-T), and RGSL1 for WMS (39,726,780 bp, C-T), ABCA13 for WYL (66,795,047 bp, T-C) (Table 1 and Table S4; Figure S8).

For eared pheasants, the results of fixed sites were: 308,394 for blue, 2,055,206 for brownW, 1,909,724 for brownC, 2,041,139 for brownE. After the intersection of SNP sites and genes, we identified three gene periods for the four populations: EARP_00011498 for blue (2,196,849 bp, G-T) and brownC (2,196,990 bp, G-A), EARP_00011465 for brownW (130,940 bp, A-T), and EARP_00013783 for brownE (1,568,598 bp, G-A) (Table 2 and Table S5; Figure S9).

In the case of Chinese pangolins, we examined 836,645 fixed sites for the SinoBurmese population, 466,184 for the mainland population, and 175,949 for the Yunnan/Guizhou population. Since no GFF files were available, we failed to construct the genetree or analyze RF distance and RectChr. Instead, we directly picked three sites from chr1 for the three groups: SinoBurmese (46,022,754 bp, G-T), mainland (50,352,879 bp, T-C), and Yunnan/Guizhou (59,347,103 bp, T-A) (Table 3 and Table S6; Figure S10).

### 3.3. The Limitation of Mitochondrial Genome Traceability

We assembled the mitochondrial genomes to assess the feasibility of using them for traceability of trafficked Tibetan macaques and eared pheasants. Haplotype networks were constructed using the circularized genomic sequences. The aligned sequence lengths for Tibetan macaques and eared pheasant were 16,545 bp and 16,687 bp, respectively. A total of 22 haplotypes were identified from the 26 Tibetan macaque individuals (HS: 3, WYL: 2, MS: 2, FJS: 3, WMS: 2, SC: 10), and 6 haplotypes were identified from the eared pheasants sampled (Blue: 3, BrownE: 1, BrownW: 1, and 1 shared by BrownW and BrownC).

From the haplotype network diagram, we found that the three eastern Tibetan macaque groups (HS, WYL, MS) were located on independent branches, indicating a high degree of divergence in their maternal lineages. In contrast, the haplotypes of the three western Tibetan macaque groups (FJS, WMS, SC) exhibited a notably scattered distribution within the network (Figure S6). Therefore, based on mitochondrial genomic data alone, Tibetan macaques can best be distinguished into eastern and western groups, with further tracing to specific populations currently unachievable. The haplotype network of eared pheasants clearly distinguished the blue and brownE groups from other populations, which corresponded to the divergence history of brown eared pheasants (Figure S7). Most brownW individuals and all brownC individuals shared one haplotype. Therefore, it was possible to trace pheasants of the blue and brownE populations using mtDNA, but not the brownW and brownC populations.

Taken together, the mitochondrial genome and haplotype network analyses indicate that mtDNA provides limited resolution for tracing different geographically distributed populations of both Tibetan macaques and eared pheasants. Although mitochondrial haplotypes successfully captured broad phylogeographic differentiation, extensive haplotype sharing and overlap among closely related populations prevented reliable assignment at finer geographical scales. These results suggest that mtDNA-based markers alone are insufficient for population-level traceability of these taxa.

## 4. Discussion

This study establishes a SNP-based genomic framework for determining the geographical origin of wildlife samples at the population level, addressing a long-standing challenge in wildlife forensic science. By integrating population genomic analyses with the development of PCR-amplifiable, population-specific SNP markers, we demonstrated that fine-scale origin assignment is achievable for multiple trafficked species. Compared with traditional mitochondrial DNA-based approaches, the proposed framework provides substantially higher resolution and greater forensic reliability, particularly for taxa with complex population structure and historical gene flow.

While species-level identification is often sufficient to confirm the illegal nature of a trafficked specimen, it provides limited information for linking seized materials to specific poaching locations, identifying trafficking routes, or supporting proportionate sentencing and deterrence [61]. In contrast, population-level traceability has the potential to accurately determine the geographic and populational origin of a confiscated species, thereby strengthening the evidentiary chain from poaching to prosecution [27,61,62].

The clear genetic structuring observed in Tibetan macaques, eared pheasants, and Chinese pangolins demonstrates that such traceable population units can be delineated even in species with recent divergence or ongoing gene flow. For example, in Tibetan macaques, our Dsuite analysis revealed asymmetric gene flow between the western FJS population and all eastern populations (Figure 4d). This pattern likely reflects male-mediated dispersal, a common feature in cercopithecine primates, combined with historical range shifts during Pleistocene climatic oscillations [33]. While such gene flow adds biological nuance to the species’ evolutionary history and may introduce shared ancestral alleles across populations, it does not undermine forensic resolution. Critically, the population-specific SNP markers we identified were selected based on fixed or near-fixed alleles within each genetic cluster (HAF ≥ 80%), ensuring that they remain diagnostic despite historical introgression. Moreover, these markers are located in genomic regions exhibiting high inter-population differentiation (e.g., genes with low RF distances), further reducing the risk of false assignment. The robust population clustering observed in PCA and admixture analyses (Figure 3) confirms that even in the presence of historical introgression, populations retain diagnostic genetic signatures suitable for forensic traceability. Nevertheless, we acknowledge that unsampled populations or cryptic gene flow could introduce uncertainty; expanding reference databases and incorporating probabilistic assignment frameworks will be important future directions.

A central finding of this study is the superior performance of nuclear SNP markers over mitochondrial DNA for fine-scale geographic assignment. Although mtDNA markers are widely used in wildlife forensics due to their high copy number and robustness to degradation, our results demonstrate that mitochondrial haplotypes capture only broad phylogeographic patterns and fail to reliably distinguish between individuals of closely related populations. Extensive haplotype sharing among western Tibetan macaque populations and between brownW and brownC eared pheasants illustrates the limitations of mtDNA in resolving recent divergence and local population structure (Figures S6 and S7). These findings are consistent with other well-documented issues associated with mtDNA, including those which focus exclusively on maternal inheritance, introgression, and incomplete lineage sorting [21]. In contrast, genome-wide SNPs reflect biparental inheritance and cumulative evolutionary history, providing the resolution necessary for population-level forensic traceability [15,63].

Beyond analytical performance, the proposed framework offers several practical advantages for real-world forensic applications. The population-specific SNP markers identified in this study are embedded within short genomic fragments, enabling reliable PCR amplification from highly degraded or trace DNA samples, which are typical of confiscated wildlife products such as processed tissues, scales, or bone fragments [24,64]. Importantly, once diagnostic loci are established, routine forensic analyses do not require whole-genome resequencing [65]. Instead, traceability can be achieved using standard PCR and Sanger sequencing or low-throughput genotyping platforms, substantially reducing analytical costs, technical complexity, and infrastructure requirements. This feature enhances the feasibility of integrating a SNP marker framework into existing wildlife forensic laboratories and law enforcement workflows.

However, implementing SNP-based forensic pipelines poses practical challenges, including high initial development costs (e.g., sequencing and validation), requirements for bioinformatics expertise, and the need for inter-laboratory validation to ensure reproducibility [66,67]. Although our minimal-marker approach enables low-cost PCR-based traceability suitable for routine forensic use, it inherently provides lower statistical confidence than likelihood-based or machine-learning assignment methods that utilize the full SNP panel. To screen for potential hybrids, we recommend pre-filtering samples with D-statistics (implemented here via Dsuite). Unsampled populations or ongoing gene flow remain inherent challenges for any fixed-allele system; our markers were derived from the most comprehensive reference datasets currently available, but blind validation on additional field or forensic samples will be essential to assess real-world robustness. Routine genotyping is more affordable (~US$8.60 per sample) but may exceed mtDNA costs in limited setting [68]. To address these, we suggest using cost-effective technologies like third-generation sequencing, forming international consortia for shared resources, and providing training programs [2,17].

In the broader context of wildlife law enforcement, integrating SNP-based evidence into judicial processes can enhance the evidentiary value in conservation-related cases. For instance, by assigning confiscated samples to specific genetic populations, SNP markers provide forensic scientists with data to infer geographic origins, thereby aiding in the identification of trafficking routes and the valuation of crimes under wildlife protection laws [61]. This genetic information, when combined with life history data and circumstantial evidence, can be presented in court to evaluate the strength of the case, similar to how DNA assignment methods are used in human forensics [22]. Future interdisciplinary collaborations between geneticists, legal experts, and policymakers could further refine these applications, promoting effective enforcement while respecting jurisdictional contexts.

Several limitations of the present study should be acknowledged. First, the SNP panels were developed using currently available population samples and do not yet encompass the full geographic ranges of the target taxa. As a result, individuals originating from unsampled or cryptic populations may not be assignable with the same level of confidence. Second, incomplete genome annotation for the Chinese pangolin constrained gene-based locus selection, and we were unable to apply the gene-tree and Robinson–Foulds distance filtering that was performed for Tibetan macaques and eared pheasants. These markers met stringent fixation thresholds (≥90% within target populations) and are supported by clear population differentiation in PCA and Admixture analyses (Figure 3). Third, it should be noted that for the eared pheasants, our framework distinguished four genetic clusters at different taxonomic levels—one species (blue eared pheasant) and three populations within its sister species (brown eared pheasant). While this demonstrates the broad applicability of the SNP-based approach across varying degrees of divergence, the traceable units are not uniformly at the population level within a single species. The framework therefore remains valid for forensic assignment among these four clusters, but readers should interpret the pheasant results in the context of their different taxonomic ranks. Fourth, although primer design was guided by stringent population-genetic criteria and built upon successful PCR validation in our prior golden snub-nosed monkey study [69], wet-lab amplification of the current primer sets on additional degraded forensic-type samples (e.g., scales, hair, processed meat) was not performed due to the extreme rarity and protected status of the original tissues. Future studies using simulated forensic materials will be essential to confirm real-world applicability.

## 5. Conclusions

In this study, we developed and validated an SNP-based genetic traceability framework for four endangered animal species in China. By leveraging genome-wide SNP data, we demonstrated that relatively small informative markers can accurately assign individuals to their geographic origins, even in species with complex evolutionary histories. Our findings highlight the robustness and general applicability of SNP-based approaches for wildlife forensic investigations. This framework provides a practical scientific basis for combating illegal wildlife trade, supporting judicial processes, and informing conservation management. With continued expansion of reference genetic databases, SNP-based traceability has strong potential to become a standardized tool in wildlife law enforcement and biodiversity conservation.

## Figures and Tables

**Table 2:** Primer information of eared pheasants.

Pop	Gene	Mutation	Left Primer	Right Primer
blue	EARP_00011498	G-T	AAAGCTTCATTCCCACAAATTG	TCCATATCACCTTGCAAAAGAA
brownC	EARP_00011498	G-A	AAAGCTTCATTCCCACAAATTG	TCCATATCACCTTGCAAAAGAA
brownW	EARP_00011465	A-T	ATAAGGGACCAATTTCAAGACC	ACCAAAAGCATGATAGTCCTTT
brownE	EARP_00013783	G-A	CTCAGAAGAAAAGAGCTATCCC	GCTGAGGATGTTTAACCAAAAG

**Table 3:** Primer information of Chinese pangolins.

Pop	Mutation	Left Primer	Right Primer
SinoBurmese	G-T	TGTCTTATGAACGTACTGTGTC	AGTGACAAAGCAACTAATCTCA
mainland	T-C	ACACTTACAGGTTTTGCATTTG	CCCCATATCTTATTGACTCTGC
Yunnan/Guizhou	T-A	ATGAAGTGTTACCTCTCCCTTA	ATAAAGCCATTCACACATCCAT

## Data Availability

Data reported in this study are available in the Genome Sequence Archive in National Genomics Data Center, China National Center for Bioinformation, Beijing Institute of Genomics, Chinese Academy of Sciences (https://ngdc.cncb.ac.cn/bioproject/browse/PRJCA026025 (accessed on 27 March 2026), GSA: CRA016435). Tibetan macaque reference material can be downloaded from NCBI (GCF_003339765.1). Eared pheasant data are available in the National Genomics Data Center (https://bigd.big.ac.cn/?lang=en (accessed on 27 March 2026), BioProject: PRJCA003284), reference material can be downloaded from GenBank database (accession number: KP259807). Chinese pangolin data are available in the CNGB Sequence Archive of China National GeneBank DataBase (CNGBdb) (accession number: CNP0001723), reference material can be downloaded from CNGBdb (BioProject: PRJCA020583).

## Supplementary Materials

The following supporting information can be downloaded at https://www.mdpi.com/article/10.3390/ani16071052/s1, Figure S1. Cross-Validation error for Tibetan macaques in Admixture analysis. CV error reaches its minimum at K = 2, suggesting that samples should be separated into 2 cluster. Figure S2. Cross-Validation error for Eared pheasants in Admixture analysis. CV error reaches its minimum at K = 4, suggesting that samples should be separated into 4 cluster. Figure S3. Cross-Validation error for Chinese pangolins in Admixture analysis. CV error reaches its minimum at K = 3, suggesting that samples should be separated into 3 cluster. Figure S4. The gene score (stand for RF distance of genes) is inversely proportional to gene length, for Tibetan macaques. Figure S5. The gene score (stand for RF distance of genes) is inversely proportional to gene length, for Eared pheasants. Figure S6. The mitochondrial haplotype network of Tibetan macaques based on mtDNA genome. All haplotype could be separated into Western and Eastern. Figure S7. The mitochondrial haplotype network of Eared pheasants based on mtDNA genome. blue Eared pheasants (blue) and brown Eared pheasants from Hebei province (brownE) can be distinguished clearly, while the haplotypes of brown Eared pheasants from Shanxi (brownC) and brown Eared pheasants from Shaanxi (brownW) are mixed. Figure S8. Gene regions containing population specific sites, for Tibetan macaques. Figure S9. Gene regions containing population specific sites, for Eared pheasants. Figure S10. Gene regions containing population specific sites, for Chinese pangolins. Table S1. Sample Information of Tibetan Macaques. Table S2. Sample Information of Eared Pheasants. Table S3. Sample Information of Chinese Pangolins. Table S4. Primer Information of Tibetan Macaques. Table S5. Primer Information of Eared Pheasants. Table S6. Primer Information of Chinese Pangolins. References [70,71,72] are cited in Supplementary Materials.
