# Supplementary material for: A Genomic Method for Combating Wildlife Trafficking: SNP-Based Traceability of Four Endangered Species in China
Source: Animals (Basel). 2026 Mar 30;16(7):1052. doi: 10.3390/ani16071052 (PMC13072273; doi:10.3390/ani16071052)
Supplement: Supplementary file 1 [file animals-16-01052-s001.zip › Figures.pdf]

A Genomic Method for Combating Wildlife Trafficking: SNP-Based Traceability of Four Endangered Species in China

Jilai Zhao [1,2,†], Xibo Wang [3,†], Yang Teng [2], Paul A. Garber [4,5], Huijuan Pan [1,*] and Jiwei Qi [2,*]

[1] School of Ecology and Nature Conservation, Beijing Forestry University, Beijing 100083, China
[2] State Key Laboratory of Animal Biodiversity Conservation and Integrated Pest Management, Institute of Zoology, Chinese Academy of Sciences, Beijing 100101, China
[3] Ministry of Education Key Laboratory for Biodiversity Science and Ecological Engineering, College of Life Sciences, Beijing Normal University, No. 19 Xin Jie Kou Wai Avenue, Beijing 100875, China
[4] International Centre of Biodiversity and Primate Conservation, Dali University, Dali 671003, China; p-garber@illinois.edu
[5] Department of Anthropology and Program in Ecology, Evolution, and Conservation Biology, University of Illinois, Urbana, IL 61801, USA
* Correspondence: phjjanine@bjfu.edu.cn (H.P.); qijiwei@ioz.ac.cn (J.Q.); Tel.: +86-10-62336801 (H.P.); +86-10-64807037 (J.Q.); Fax: +86-10-64807099 (J.Q.)
† These authors contributed equally to this work.

Supplementary Figures

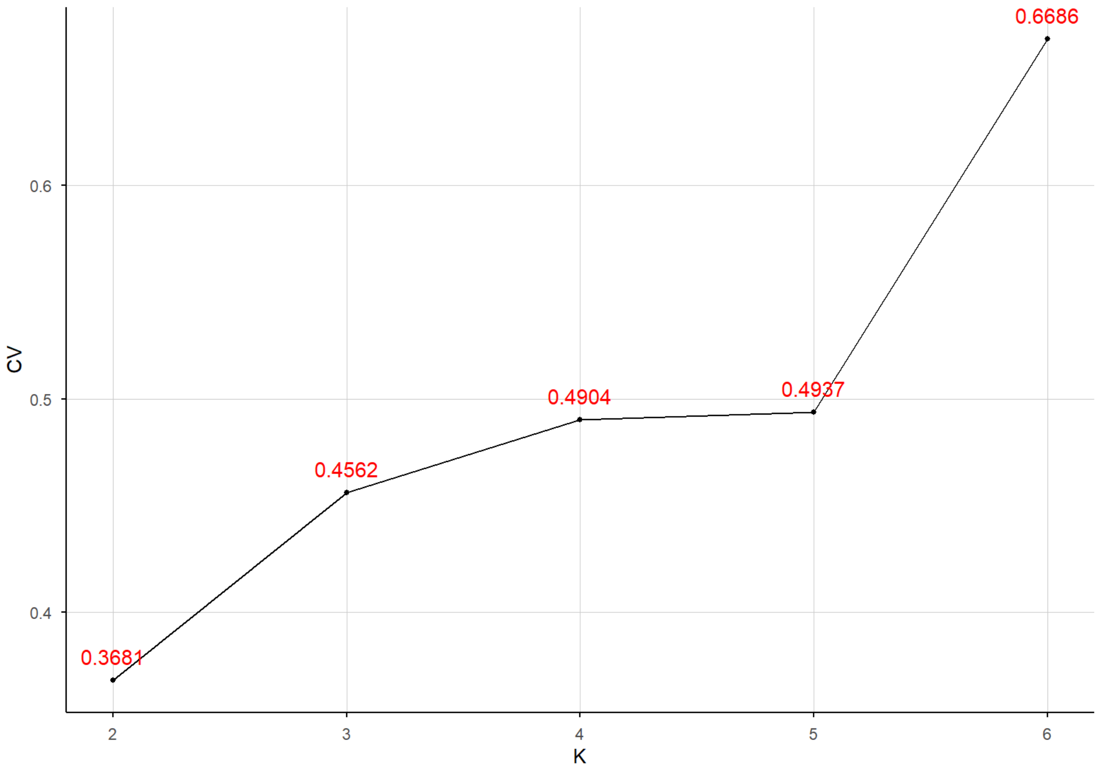

Figure S1. Cross-Validation error for Tibetan macaques in Admixture analysis. CV error reaches its minimum at K=2, suggesting that samples should be separated into 2 cluster.

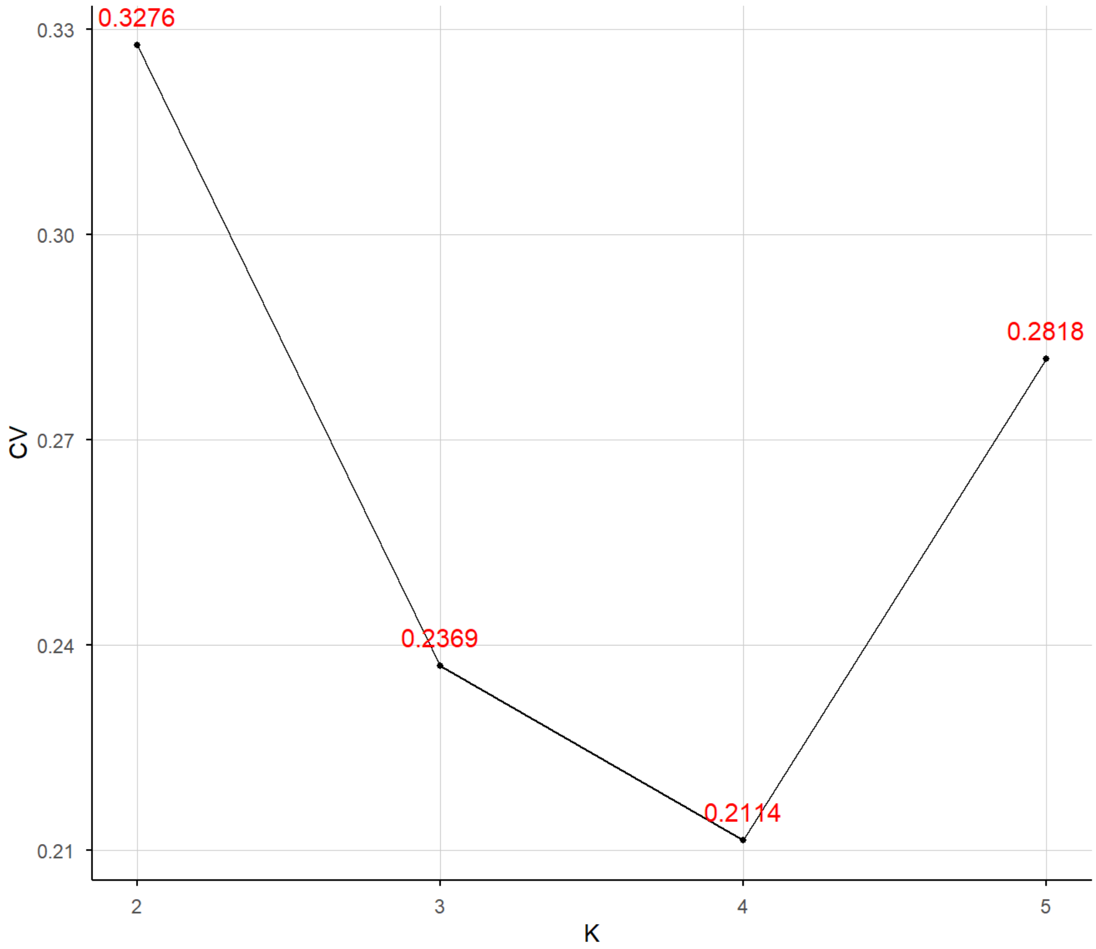

Figure S2. Cross-Validation error for Eared pheasants in Admixture analysis. CV error reaches its minimum at K=4, suggesting that samples should be separated into 4 cluster.

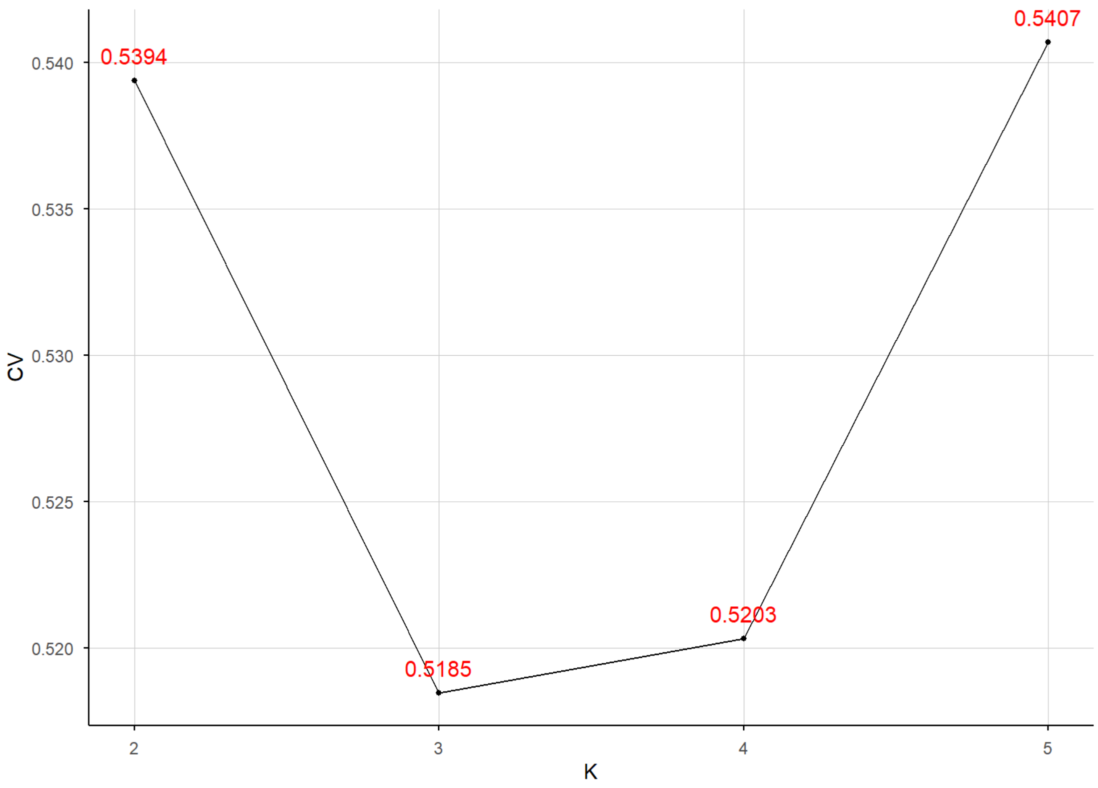

Figure S3. Cross-Validation error for Chinese pangolins in Admixture analysis. CV error reaches its minimum at K=3, suggesting that samples should be separated into 3 cluster.

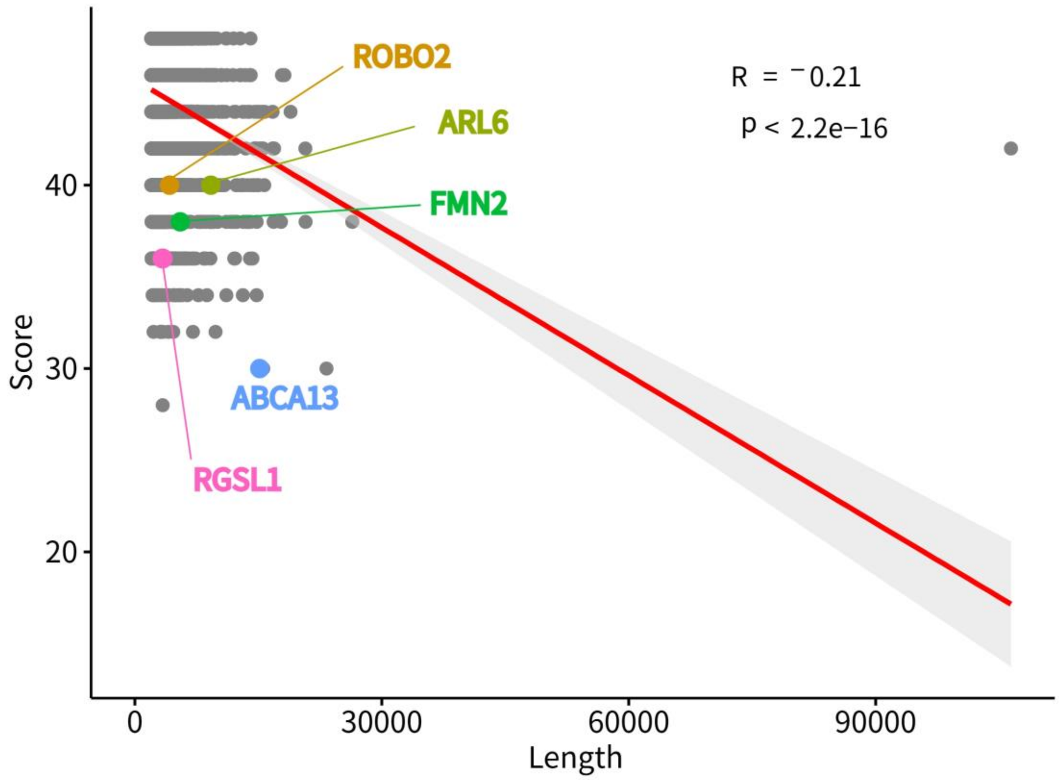

Figure S4. The gene score (stand for RF distance of genes) is inversely proportional to gene length, for Tibetan macaques.

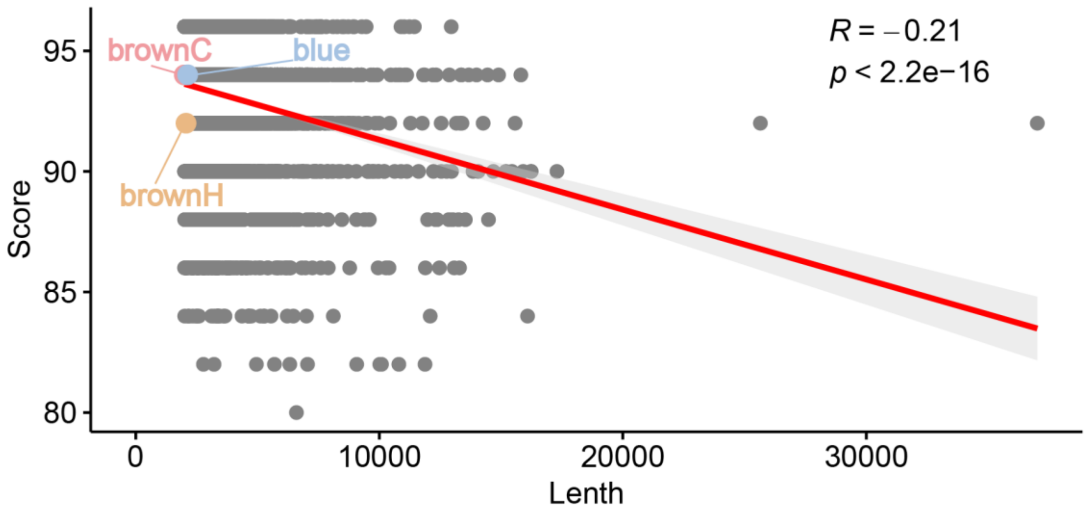

Figure S5. The gene score (stand for RF distance of genes) is inversely proportional to gene length, for Eared pheasants.

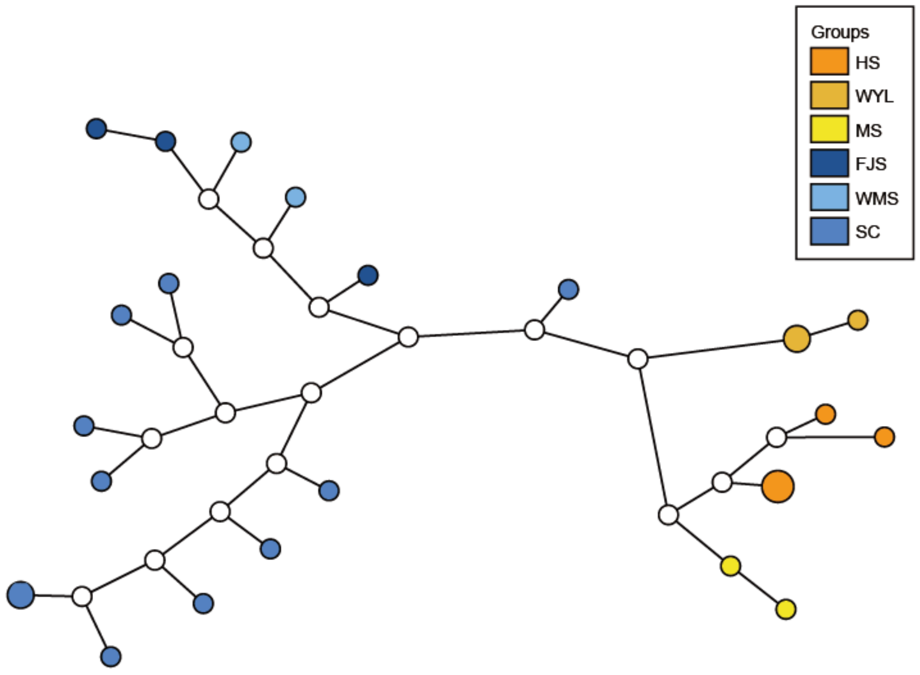

Figure S6. The mitochondrial haplotype network of Tibetan macaques based on mtDNA genome. All haplotype could be separated into Western and Eastern.

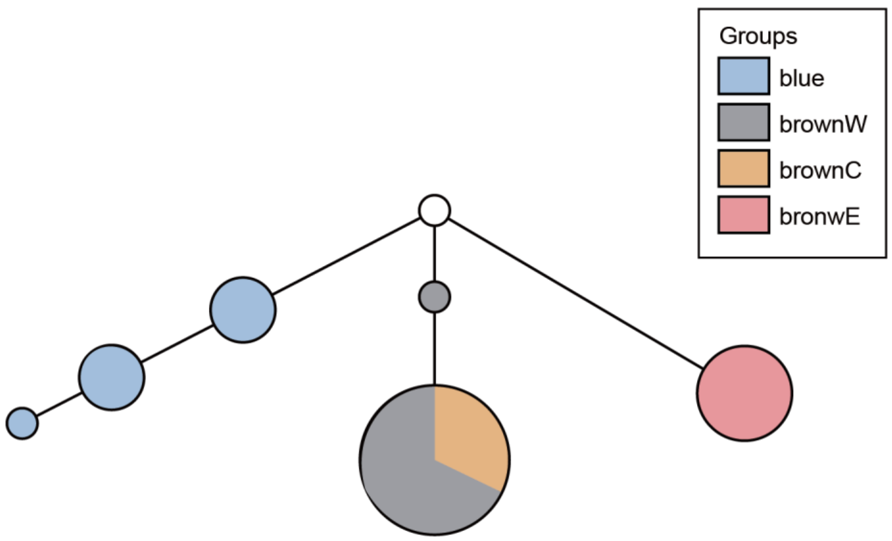

Figure S7. The mitochondrial haplotype network of Eared pheasants based on mtDNA genome. blue Eared pheasants (blue) and brown Eared pheasants from Hebei province (brownE) can be distinguished clearly, while the haplotypes of brown Eared pheasants from Shanxi (brownC) and brown Eared pheasants from Shaanxi (brownW) are mixed.

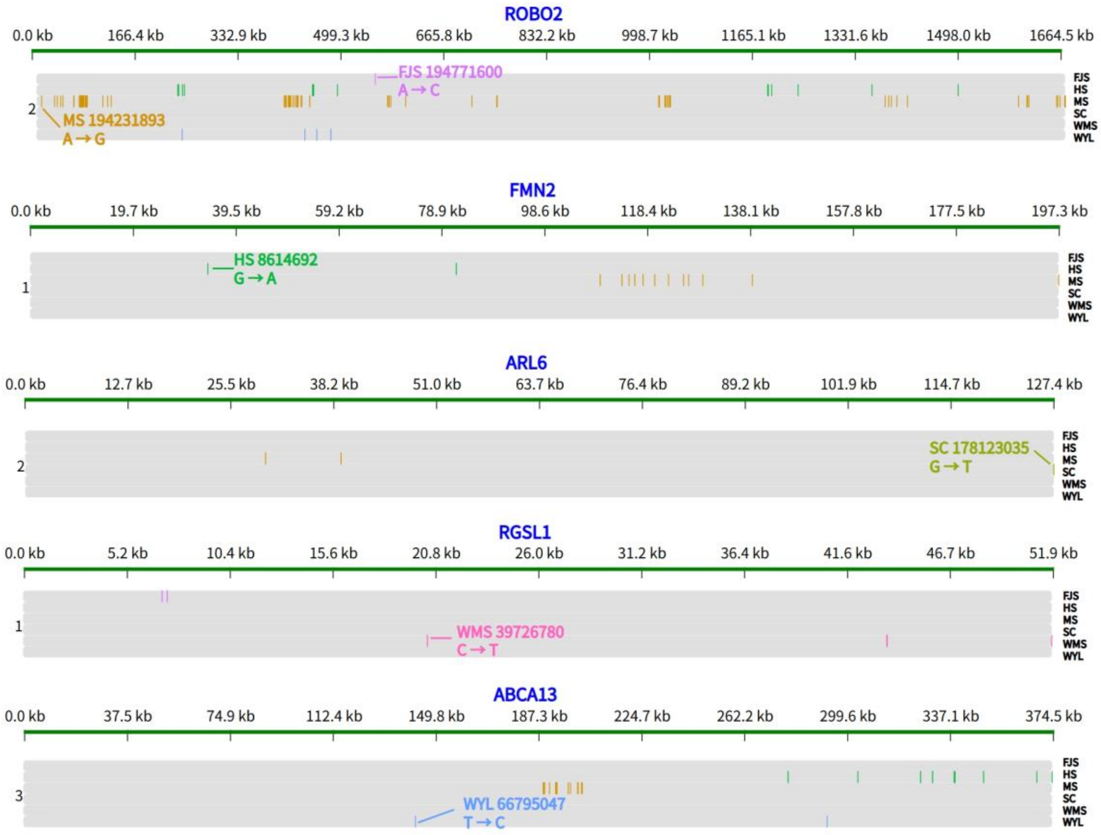

Figure S8. Gene regions containing population specific sites, for Tibetan macaques.

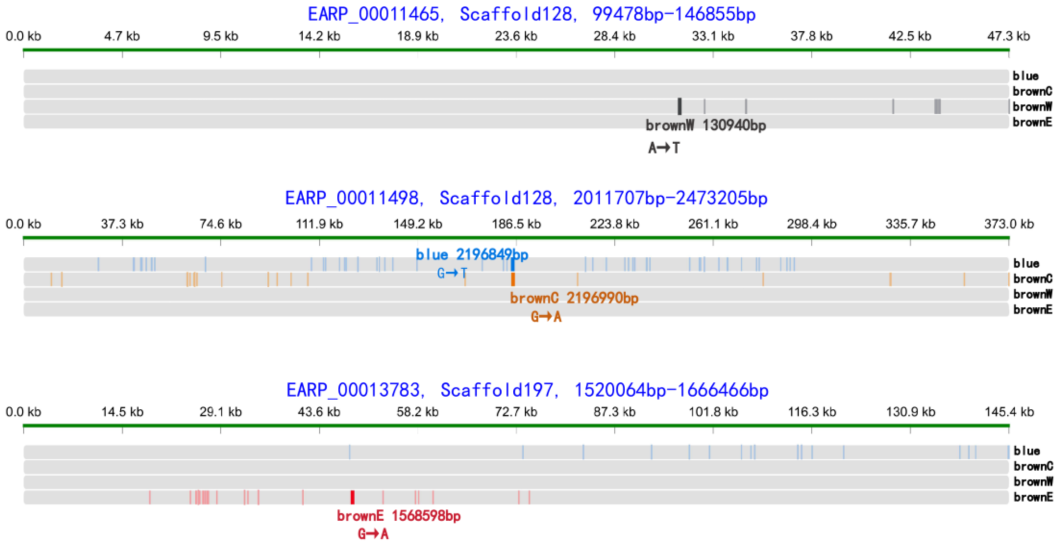

Figure S9. Gene regions containing population specific sites, for Eared pheasants.

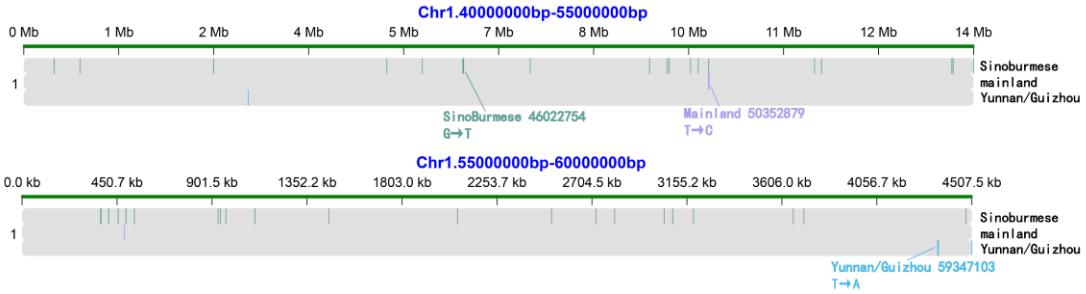

Figure S10. Gene regions containing population specific sites, for Chinese pangolins.
